# 62 Plasma Inclusive Resuscitation Effects on Extracellular Vesicles and Tissue Factor in Major Thermal Injury Patients

**DOI:** 10.1093/jbcr/iraf019.062

**Published:** 2025-04-01

**Authors:** Meghan Fondakowski, Thomas Orfeo, Samuel DiPasquale, Matthew Gissel, Melissa McLawhorn, Anthony Pusateri, Lauren Moffatt, Jeffrey Shupp, Maria Cristina Bravo

**Affiliations:** University of Vermont; University of Vermont; University of Vermont; University of Vermont; MedStar Health Research Institute; Naval Medical Research Unit San Antonio; MedStar Health Research Institute; MedStar Washington Hospital Center; University of Vermont

## Abstract

**Introduction:**

Major thermal injury triggers significant local and systemic responses, one of which is the release of membrane-derived particles (extracellular vesicles (EVs)) from damaged or stressed cells. Microvesicles (MVs) (EVs with diameters ≥ 100 nm) reflect protein and lipid characteristics of the plasma membranes of the cells from which they originate. Functional tissue factor (TF), a plasma membrane bound initiator of coagulation, has been identified in plasmas from burn patients. It has been hypothesized that elevated levels of MV associated TF may contribute to coagulopathies observed with severe burn injury. The potential of administered fresh frozen plasma (FFP) to alter MV levels and their associated TF dependent procoagulant potential has not been explored.

**Methods:**

Patients (n=23) with varying burn severity (TBSA: 40 ± 19%) were enrolled prospectively in an IRB approved study. Blood was collected and platelet poor plasma prepared prior to administration of the first transfusion unit (BD1: time from injury 383 ± 249 min) and immediately following (BD2: 25 ± 11 min post BD1). Plasmas from patients or administered FFP units were thawed in the presence of corn trypsin inhibitor (blocks FXII-initiated coagulation), EV populations isolated via gel filtration, and vesicle numbers and size distribution quantified using a pressurized nanopore system. Functional TF concentrations in the EV isolates were assessed using an assay in which the rate of activation of FX to FXa via FVIIa is proportional to [TF]. Rates of FX activation by EV isolates were translated into TF equivalents using a standard curve constructed using a commercial TF of known concentration. Data are presented as mean ± SD; paired t-tests were used to compare findings across sampling times (significance: p ≤ 0.05).

**Results:**

Total MV concentrations were 1.6 ± 1.8 *10^11 particles/mL at BD1 and did not differ from that observed at BD2 (1.1 ± 1.1 *10^11 particles/mL). The MV fraction of all EVs at BD1 (14 ± 8%) did not differ from that at BD2 (13 ± 8%). Similarly, functional TF concentrations in patient plasmas did not alter after administration of a FFP unit (BD1: 65 ± 70 fM; BD2: 72 ± 78 fM). MV and TF levels in administered FFP units ([MV] = 0.5 ± 0.6 *10^11 particles/mL, [TF] = 10 ± 7 fM) were lower than those seen in patient plasmas (p ≤ 0.05). Concentrations of MVs and TF correlated with burn severity (r=0.47 for both), and each other (r=0.52).

**Conclusions:**

Following burn injury, there are elevated levels of MVs and TF-bearing MVs that positively correlate with TBSA. Administration of FFP does not alter the amount of TF-bearing MVs following major thermal injury.

**Applicability of Research to Practice:**

The data indicate that initial FFP administration does not exacerbate the increased levels of circulating EVs and TF that characterize severe burn injury.

**Funding for the Study:**

USAMRAAA - BA190086

